# Challenges for Clinical Drug Development in Pulmonary Fibrosis

**DOI:** 10.3389/fphar.2022.823085

**Published:** 2022-01-31

**Authors:** Eric S. White, Matthew Thomas, Susanne Stowasser, Kay Tetzlaff

**Affiliations:** ^1^ Boehringer Ingelheim Pharmaceuticals, Inc., Ridgefield, CT, United States; ^2^ Boehringer Ingelheim Pharma GmbH & Co. KG, Biberach, Germany; ^3^ Boehringer Ingelheim International GmbH, Ingelheim am Rhein, Germany; ^4^ Department of Sports Medicine, University of Tübingen, Tübingen, Germany

**Keywords:** lung disease, interstitial, clinical trial, drugs, fibroblast, pharmaceutical research, pharmacology

## Abstract

Pulmonary fibrosis is a pathologic process associated with scarring of the lung interstitium. Interstitial lung diseases (ILDs) encompass a large and heterogenous group of disorders, a number of which are characterized by progressive pulmonary fibrosis that leads to respiratory failure and death. Idiopathic pulmonary fibrosis (IPF) has been described as an archetype of progressive fibrosing ILD, and the development of pirfenidone and nintedanib has been a major breakthrough in the treatment of patients with this deadly disease. Both drugs principally target scar-forming fibroblasts and have been shown to significantly slow down the accelerated decline of lung function by approximately 50%. In addition, nintedanib has been approved for patients with other progressive fibrosing ILDs and systemic sclerosis-associated ILD. However, there is still no cure for pulmonary fibrosis and no meaningful improvement of symptoms or quality of life has been shown. Advancement in research, such as the advent of single cell sequencing technology, has identified additional pathologic cell populations beyond the fibroblast which could be targeted for therapeutic purposes. The preclinical and clinical development of novel drug candidates is hampered by profound challenges such as a lack of sensitive clinical outcomes or suitable biomarkers that would provide an early indication of patient benefit. With the availability of these anti-fibrotic treatments, it has become even more difficult to demonstrate added efficacy, in particular in short-term clinical studies. Patient heterogeneity and the paucity of biomarkers of disease activity further complicate clinical development. It is conceivable that future treatment of pulmonary fibrosis will need to embrace more precision in treating the right patient at the right time, explore novel measures of efficacy, and likely combine treatment options.

## Introduction

The last decade has been a game changer for idiopathic pulmonary fibrosis (IPF) from a research as well as from a clinical perspective. A shift of understanding of the pathogenesis from a chronic inflammatory to an epithelial driven disorder resulting from repetitive alveolar epithelial injury and aberrant repair (Selman et al., 2001; King et al., 2011) as well as a refinement of diagnostic criteria for IPF (Raghu et al., 2012) finally enabled a breakthrough in the treatment of patients with this devastating disease with the approval of nintedanib and pirfenidone (Richeldi et al., 2011; Raghu et al., 2012; King et al., 2014).

IPF is the most common form of idiopathic interstitial pneumonia within a large and heterogenous group of disorders referred to as interstitial lung diseases (ILD) (Travis et al., 2013). IPF is, by definition, a chronic progressive fibrosing ILD characterized by inexorable worsening of symptoms, lung function and early mortality (Raghu et al., 2018) and is considered the prototypical progressive fibrotic ILD. However, such longitudinal disease behaviour (termed “progressive fibrotic phenotype”) is not restricted to IPF but applies to other ILDs with even greater heterogeneity compared to IPF (Travis et al., 2013; Wells et al., 2018). It is postulated that progressive fibrosing ILDs also share similarities in pathogenic mechanisms implicated in fibrotic remodeling (Wollin et al., 2019). Consequently, pirfenidone and nintedanib were separately investigated in a basket approach, similar to oncology, including non-IPF patients with differing underlying etiologies but a common progressive fibrotic clinical disease course (Flaherty et al., 2019; Behr et al., 2021), further supporting the concept of the progressive fibrotic phenotype and leading to the approval of nintedanib in patients with progressive fibrosing interstitial lung disease (Brown et al., 2020).

Undoubtedly these advances and, specifically, the approvals of antifibrotic drugs for the treatment of (idiopathic) pulmonary fibrosis have fuelled basic, translational and clinical research and drug development in this field. However, key challenges will have to be tackled across disciplines in the next decade to ultimately embark on a path of patient-centricity and precision medicine in drug development and to provide benefit to patients beyond “just” slowing lung function decline. These include identification of patients earlier in their disease course to ultimately prevent development of or even reverse fibrosis, validation of prognostic and predictive biomarkers to enable the right treatment for the right patient at the right time, developing better disease models, clinical outcome parameters and, ultimately, targeting the mechanisms involved in lung repair and regeneration.

This review aims to shed light on some of these key challenges from an industry research and development perspective.

## Evolving Mechanisms of Pulmonary Fibrosis

Both pirfenidone and nintedanib—the current standard of care for medications for idiopathic and other forms of pulmonary fibrosis—were borne of the desire to inhibit aberrant fibroblast activity. Fibroblast populations are responsible for providing structure to organs, so are thus an essential part of the repair process following damage. Fibroblast proliferation, migration and transition toward myofibroblasts, which preferentially deposit collagen to form scar tissue, are healthy processes when controlled to be transient (Ushakumary et al., 2021). However, the dysregulation of these processes leads to uncontrolled fibrogenesis—a fundamental driver or “master mechanism” of pulmonary fibrosis. Anti-fibrotic therapies address elements of these pathologic pathways through inhibition of fibroblast-centric growth factors (Conte et al., 2014; Wollin et al., 2014)

**FIGURE 1:**
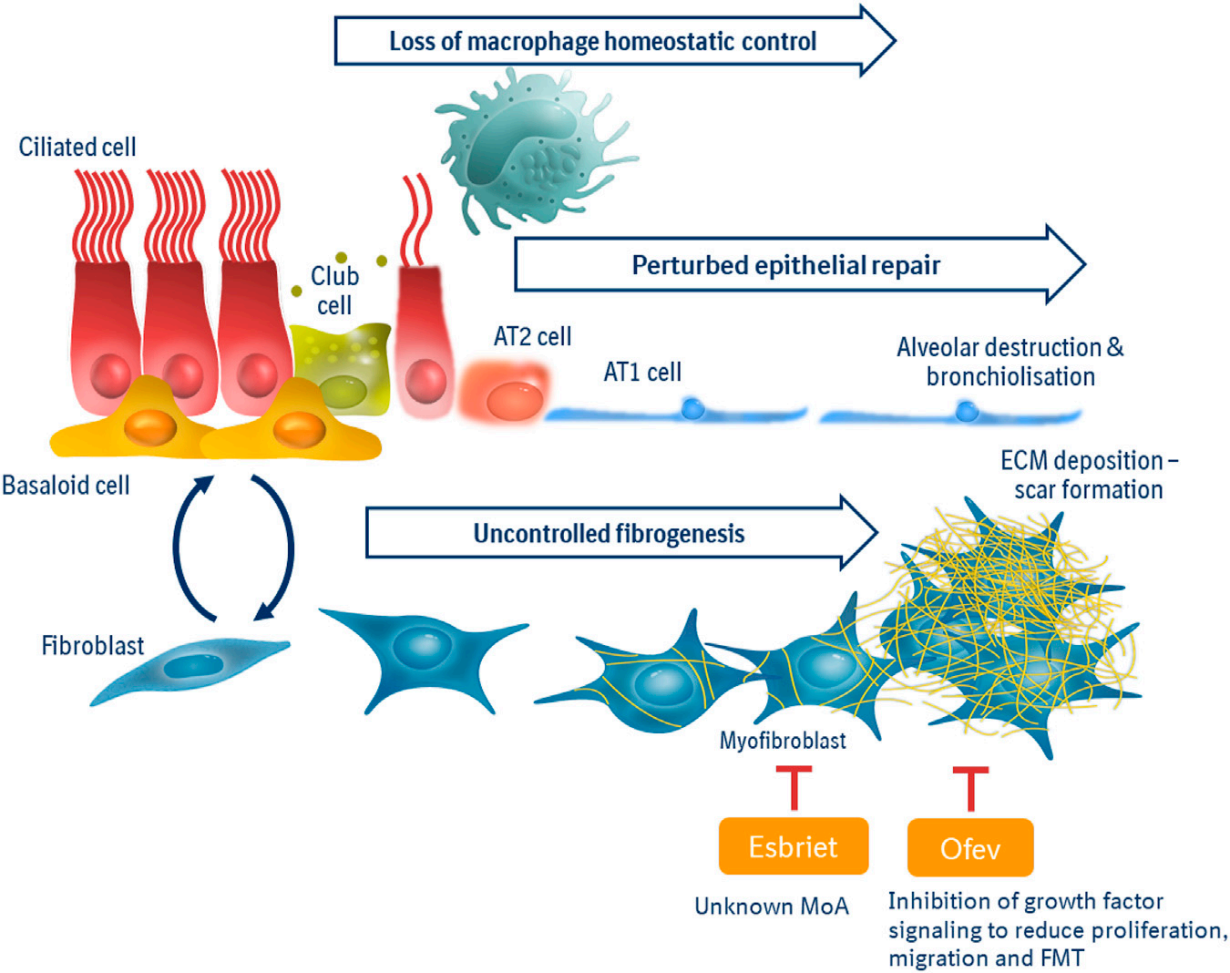
Master Mechanisms of the immunofibrotic niche. Pathologic drivers in pulmonary fibrosis can be viewed as three “master mechanisms”: Uncontrolled fibrogenesis, driven by pro-fibrotic growth factors and tissue stiffness, is the current point of intervention for standards of care “anti-fibrotics.” Perturbed epithelial repair is due to dysregulated/transitional small airway and/or alveolar progenitor populations, leading to senescence, impaired barrier function and poor gas exchange. Loss of macrophage homeostatic control is characterized by an imbalance of pro-fibrotic vs. pro-repair macrophage phenotypes to resulting in less efferocytosis and heighted inflammation within the lung. The immunofibrotic niche is interconnected, where macrophage activity and phenotype can be influenced by senescent fibroblasts; aberrant signals from epithelium promote fibrosis and scar-forming fibroblasts hinder effective epithelial repair.

The advent of single cell seq technologies have greatly expanded our view of pathologic processes to reach beyond the fibroblast (Habermann et al., 2019; Reyfman et al., 2019; Adams et al., 2020). We now recognize profound changes in epithelial stem cell populations which drive perturbed barrier repair and impaired lung function. Disease-specific “aberrant basaloid” cells may be derived from dysregulated secretory cell phenotypes or dysfunctional/transitional alveolar type 2 populations (Adams et al., 2020; Jaeger et al., 2020; Strunz et al., 2020). Signaling thought to drive the perturbed epithelial repair include oxidative and endoplasmic reticulum stress (Burman et al., 2018; Otoupalova et al., 2020). An additional “master mechanism” for consideration is the phenotypic alteration to macrophage populations driving a loss of homeostatic control within a fibrotic lung. Although long associated with progressive pulmonary fibrosis (Keogh and Crystal, 1982), early attempts to modulate macrophage migration into the lung failed (Raghu et al., 2015)—likely due to the translational limitations of previously dominant murine M1/M2 hypothesis of macrophage pathobiology. Triggered, again, by SCseq studies, we have now expanded our knowledge of macrophages and fibrosis to reveal a key role for efferocytosis in the effective (anti-fibrotic) repair of the lung (Morse et al., 2019; Gerlach et al., 2021). Furthermore, biomarker evidence of senescence in pulmonary fibrosis (Ryu et al., 2017) is being unraveled across “master mechanisms”—linked to dysregulated fibroblasts, aberrant epithelial stem cell populations and as drivers of a pro-fibrotic macrophage phenotype (Álvarez et al., 2017; DePianto et al., 2021). Furthermore, we are now understanding more than ever that to consider any cell population in isolation is to miss the (mis-)communication between different populations—cell types which collude to drive fibrosis relentlessly forward (Raredon, Systems Biol 2020; Le et al., 2021). Preclinical research into the “immunofibrotic niche” (summarized in Figure 1) has opened new avenues for drug discovery.

We now look to promote a homeostatic balance of macrophages, rejuvenate small airway and alveolar epithelial progenitor populations and correct the mutual dysfunctional crosstalk between epithelium and underlying fibroblasts. Yet the ambition to explore novel targets within the immune fibrotic niche preclinically will bring even greater challenges as they are translated to the clinic—requiring an ever greater degree of therapeutic precision.

## Challenges in Preclinical Research of Emerging Therapeutics

Anti-fibrotic therapeutics were validated with *in vitro* and *in vivo* systems focused upon fibroblast biology. Fibroblasts stimulated with platelet-derived growth factor (PDGF) will proliferate, or with transforming growth factor (TGF)ß transition to scar-forming myofibroblasts. Bleomycin-induced fibrosis in mice/rats model inflammatory, then fibrotic, changes followed by resolution waves of pathobiology—yet have shown limited translational value (Moeller et al., 2008). It is interesting to note that although fibroblasts remain the principal target cell population of both nintedanib and pirfenidone, this does not preclude some effects on other cell populations—either directly or as a consequence of altering the dynamics within the immune-fibrotic niche (Huang et al., 2017).

**FIGURE 2:**
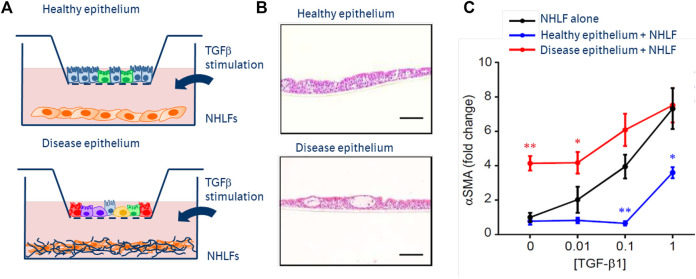
Next generation co-culture assays of epithelial:fibroblast crosstalk. **(A)** Schematics of epithelial cells, cultured in inserts at air-liquid interface from primary basal epithelium isolated from healthy or fibrosis patient lungs. Normal healthy lung fibroblasts (NHLFs) co-cultured in the below wells—stimulated with increasing concentrations of TGFß. **(B)** H&E images of healthy vs diseased (fibrotic) epithelium, showing fewer ciliated cells and more secretory populations. **(C)** Mono-culture NHLF response to TGFß dose response (black), showing increased a-smooth muscle actin—an indicator of fibroblast to myofibroblast transition. Fibrotic epithelium promotes fibroblast transition in the absence of TGFß (red). Healthy epithelial co-culture protects fibroblasts from pro-fibrotic effects of TGFß.

The next generation of novel therapeutics need innovative *in vitro* and *in vivo* systems. By enriching the environment in which primary co-cultured cells act and react *in vitro*, including stimuli which are more representative of the pathophysiologic situation, we increase the likelihood of observing a difference between healthy and diseased cells. Once observed, this ‘differential patient biology’ can form the basis of novel therapeutic discovery. For example, we can observe a reciprocal influence of primary small airway epithelial cells (SAECs), grown at air-liquid interface, together with fibroblasts (see Figure 2). When derived from healthy donors, epithelial cells exert a protective effect on fibroblasts challenged with a fibrosis-driving growth factor (TGFß). However, if derived from pulmonary fibrosis patient donors or conditioned with stimuli from fibrosis patients, SAECs can drive fibrotic transition in fibroblasts in the absence of stimulation (Le et al., 2020).

*In vivo* models, although no longer the source of novel targets, are nevertheless fundamental to understanding pharmacokinetic (PK) vs target engagement (TE) vs pharmacodynamic (PD) relationships of drug candidates. Yet, we strive to enhance the translational relevance of PD measures—functional measures or biomarkers—to capture more elements of the immune-fibrotic niche. Bleomycin models can be enhanced by using older mice, thus partially negating the transient nature of the fibrosis observed, or by using a model of repetitive instillation to create a more durable fibrotic response (Redente et al., 2021). Furthermore, microCT measures have been developed to better mimic diagnostic measures from the clinic (Ruscitti et al., 2017). Alternative models have been developed which better reveal the perturbed epithelial elements of pulmonary fibrosis, such as the model of SPC mutation, which creates an alveolar type 2 cell-driven fibrotic response (Nureki et al., 2018). Few rodent models have come closer to patient biology than that which uses a combination of humanized mice, low-dose bleomycin and adoptively transferred patient epithelial cells reproduce the lung reticulation patterns so characteristic of usual interstitial pneumonia (UIP) seen in IPF (Jaeger et al., 2020).

Novel *in vitro* and *in vivo* systems can now be assessed for greater translational relevance by the comparative computational analysis of gene expression changes in an assay vs patient material. Thus, individual pathways/processes, and the impact of test compounds upon them, can be effectively matched to the appropriate assay. It is with preclinical systems such as these that we can create first evidence in support of either combination or differentiation for a next generation therapeutic with standard of care anti-fibrotics. Through these technological breakthroughs, it is now possible to employ precision pharmacology, and thus set the stage for clinical assessment of precision therapies.

## Challenges in Clinical Development of Emerging Therapeutics

### Enrichment Strategies and Efficacy Endpoints

The widespread regulatory approval of nintedanib and pirfenidone to reduce the rate of decline in a key measure of lung function, forced vital capacity (FVC), is seen as a major advance for the treatment of patients with IPF (and, in the case of nintedanib, for patients with systemic sclerosis-associated ILD and with chronic fibrosing ILDs with a progressive phenotype). However, this success is also accompanied by new challenges for drug developers. In the ideal clinical trial, all patients enrolled in the trial would experience the event the study drug is designed to prevent/treat, such that patients receiving active drug will demonstrate clear efficacy as compared to placebo. This is especially important in conditions which take a long time to manifest or in which relatively few patients experience the event. Indeed, fibrotic ILDs often progress slowly; thus, in some patients it may take years to see significant declines in lung physiology or increased fibrosis on high-resolution computed tomography (HRCT) scans, and clinical trials often require primary endpoints to be measured after a minimum of 12 months to allow sufficient patients to demonstrate progression. Yet even in such circumstances, a proportion of patients will not demonstrate significant loss of lung function. As an example, in the pivotal INPULSIS trials of nintedanib in patients with IPF, 15% of placebo-treated patients demonstrated either no decline or an improvement in FVC over the 52-week trial period (Brown et al., 2019). Similar findings were observed in the INBUILD trial of nintedanib in patients with progressive fibrotic ILDs (Maher et al., 2021).

For fibrotic ILDs, a current challenge is identifying which patients will develop progression over a relatively short period of time or are at increased risk for exacerbations or death. This is critical not only for planning of clinical trials but also for clinical care; patients likely to experience faster progression or at risk for adverse outcomes might be prioritized on transplant lists or followed more closely in the clinic. Efforts to develop prognostic information in patients with fibrotic ILDs have intensified greatly over the recent past, with numerous investigators evaluating a panoply of potential biomarkers. Such biomarkers may be genetic (Fingerlin et al., 2013; Stuart et al., 2015; Herazo-Maya et al., 2017), metabolomic (Zhao et al., 2017; Nambiar et al., 2021), circulating soluble factors in plasma or serum (Rosas et al., 2008; Makiguchi et al., 2016; Maher et al., 2017; Organ et al., 2019), cell-based (Morse et al., 2019; Karampitsakos et al., 2021), image-based (Justet et al., 2017; Salisbury et al., 2017; Win et al., 2018; Derlin et al., 2021), or based on clinical characteristics (Ley et al., 2012; Ryerson et al., 2014; Morisset et al., 2017). While these efforts are laudable and show promise, none is yet sufficiently validated to be used in clinical practice. For clinical trial planning, enrichment strategies based on biomarkers are similarly not well validated, yet are desperately needed. One possible exception to the current lack of enrichment biomarkers is the imaging pattern of usual interstitial pneumonia (UIP) seen on HRCT scanning. UIP is the radiologic and histopathologic pattern associated with IPF, as well as other ILDs that may demonstrate more rapid progression in a similar fashion. Indeed, in the INBUILD trial of nintedanib for treatment of patients with non-IPF progressive fibrotic ILDs of varying causes, patients with an HRCT pattern of UIP demonstrated similar declines in FVC over 52 weeks as has been seen in prior IPF trials (Flaherty et al., 2019).

But for which outcome should we be trying to enrich? Historically, registrational trials for both nintedanib and pirfenidone in patients with IPF were predicated on a primary endpoint of annualized rate of decline of FVC (ml/year) or change in FVC from baseline (ml), supported by additional secondary outcomes such as a trend for reduced mortality, further strengthening FVC as a valid surrogate endpoint. Subsequent studies in IPF, as well asother fibrosing ILDs, have similarly focused on the rate of FVC decline given the precedent set by nintedanib and pirfenidone. Certainly, slowing the rate of decline in lung function for patients with IPF and other progressive ILDs is a worthy goal; data suggest that rate of decline of lung function, both FVC and diffusing capacity for carbon monoxide (DLCO), correlates with time to mortality (Collard et al., 2003; Latsi et al., 2003). This correlation between lung function decline and mortality bolstered active discussions with authorities, in particular the US FDA, which finally accepted FVC as a surrogate outcome marker for mortality in IPF; the acceptance of FVC decline was also based on review of study data from both the nintedanib and pirfenidone clinical development programs (Karimi-Shah and Chowdhury, 2015). Now, evolving real-world data suggest that patients with IPF receiving antifibrotic medication demonstrate better survival than those not treated (Guenther et al., 2018; Behr et al., 2020), lending credence to the concept that FVC decline is an appropriate surrogate for mortality (Karimi-Shah and Chowdhury, 2015). However, the relationship between lung function decline and mortality is indirect and doesn’t capture the impact of progressive ILDs on patients’ quality of life, symptoms and/or disability. Further, clinical experience tells us that the rate of decline of lung function in IPF and other ILDs is not necessarily linear over time; therefore, careful thought must be given to identifying, validating, and qualifying novel endpoints to determine the impact of new therapeutics for these patients.

### Trial Design Challenges

Both nintedanib and pirfenidone utilized classical drug development approaches with large and long phase II dose-ranging studies. For example, the trial providing proof of clinical concept for nintedanib was a dose-ranging study exploring four doses of nintedanib versus placebo that randomized 432 patients with IPF to treatment over 1 year (Richeldi et al., 2011). This study was followed by two large placebo-controlled confirmatory phase III studies comprising a total of 1,066 patients with IPF randomized to 52 weeks of treatment with nintedanib or placebo to establish pivotal efficacy and safety data for submission (Richeldi et al., 2014).

However, newer drug candidates will be required to utilize different clinical development approaches, for a variety of reasons. First, IPF (and the majority of other progressive fibrosing ILDs) are rare-to-uncommon diseases and, thus, the patient pool available for inclusion in clinical studies in these indications is limited. Coupled with increased drug development efforts in pulmonary fibrosis by numerous companies, several drug candidates are currently being investigated in phase II and phase III studies (Spagnolo et al., 2020) which leadsto competition for patients among potential trials. One potential solution to this problem was recently demonstrated by the successful conduct of the INBUILD trial, in which patients with various fibrosing ILDs (excluding IPF) who all shared a common phenotype of progressive disease were randomized to receive placebo or nintedanib (Flaherty et al., 2017). This “basket” study showed that in patients with a UIP-like pattern on HRCT but an underlying diagnosis other than IPF, nintedanib has a similar effect on slowing the rate of decline in FVC compared to what was seen in prior IPF trials (Flaherty et al., 2019). Moreover, patients with fibrosing ILD with other radiologic fibrotic patterns, but still with a prior history of disease progression, equally benefitted from nintedanib (Flaherty et al., 2019). This suggests that pooling patients who have different disease etiologies a but similar longitudinal clinical behavior may be an appropriate enrichment strategy, although this concept remains a source of debate (Wells et al., 2018; Wolters et al., 2018).

Second, accelerated new drug development timelines are necessary due to pragmatic considerations for our patients as well as costs: a long phase II study with endpoints measured 1 year after study initiation is no longer sustainable for patients or sponsors. However, the ability to demonstrate a statistically significant treatment difference in rate of FVC decline at a timepoint earlier than 52 weeks is dependent upon the degree of patient heterogeneity within a cohort and the variability inherent in lung function testing. As a result, increased sample sizes are required to achieve the power needed to detect significant differences in rate of FVC decline. Interestingly, retrospective analyses of nintedanib and pirfenidone clinical development programs suggest that a separation in the rate of decline of FVC with antifibrotic treatment may be apparent after 26 weeks. Some sponsors have recently reported results demonstrating treatment effects in phase II trials of patients with IPF after only 12 weeks (Maher et al., 2018; Palmer et al., 2018). Ziritaxestat, for example, an autotaxin (an enzyme catalyzing lysophosphatidylcholine into lysophosphatidic acid [LPA]) inhibitor demonstrated favorable effects on mean change from baseline in FVC after 12 weeks, with an increase of 8 ml in the active group versus a reduction of 87 ml on placebo (Maher et al., 2018). Similarly, treatment of antifibrotic-naïve patients with IPF with the LPA receptor antagonist BMS-986020 also resulted in a slowing of the rate of decline in FVC compared to placebo-treated patients, although the results were not statistically significant until 26 weeks (Palmer et al., 2018).

Finally, new drug candidates for patients with IPF or other progressive fibrosing ILDs can no longer ethically be compared to true placebo, as standard of care therapies (with their associated adverse effects) now exist for these patients. This adds further complexity and challenges on clinical trial design: any treatment (or adverse) effects seen for newer therapeutics could be attributed to the investigational compound or the standard background therapy; the room for improvement in lung function on top of standard of care is variable and limited and affecting sample size; patient populations will be heterogenous including all-comers, i.e., patients without background antifibrotic standard of care (either naïve to antifibrotic treatment or having stopped previous standard of care for tolerability or effectiveness reasons) and patients with concomitant approved antifibrotic treatments. Moreover, in the future, newer therapeutics may be designed to improve the lives of patients with IPF and other fibrosing ILDs as measured by different endpoints (e.g. quality of life, imaging, or other biomarkers of disease activity), which may be difficult to interpret in the face of combination therapy.

### Approaches to Increase Trial Efficiency

Given the challenge of recruiting enough patients in a timely fashion to identify drug efficacy in pulmonary fibrotic diseases, drug developers may seek to take advantage of a number of methods to speed development program efficiency. Such methods may involve innovative statistical modeling, novel trial design approaches, biomarker enrichment strategies, or some combination thereof. One example of innovative statistical modeling, called Bayesian dynamic borrowing (Dron et al., 2019), utilizes data from historical control cohorts to decrease the required number of control subjects, in some cases by up to 80%, without compromising statistical results (Dron et al., 2019). Similarly, efficiency in clinical development programs may be realized through the use of “master protocol” designs, such as confirmatory basket, umbrella and platform trials (Angus et al., 2019; Collignon et al., 2020). For example, adaptive platform trials (APTs) are perhaps least similar to the gold-standard randomized control trial, in that they study multiple interventions in a single disease (or condition) in a perpetual manner, with interventions allowed to enter or leave the platform on the basis of a decision algorithm’ (Woodcock and LaVange, 2017) rather than compare a single intervention with a control group. This approach allows for multiple treatment comparators to enter or leave a trial without having to halt the study or pause other investigative arms. Moreover, as the name implies, APTs may utilize within-trial adaptations such as response-adaptive randomization that allow for arms of a trial that are showing promise to preferentially recruit subsequent subjects, or rules that allow for transitioning from one phase to the next during the course of a trial (Angus et al., 2019). Finally, utilization of various biomarkers to allow early read-out of efficacy signals or to enrich trials for patients who will demonstrate progression over the course of a trial could also potentially improve study efficiency. While there are no universally agreed-upon biomarkers to achieve this goal, there has been substantial progress recently to detect blood biomarkers predictive of disease progression in IPF, such as markers of epithelial cell dysfunction and extracellular matrix remodeling: serum levels of surfactant protein A (SP-A) and D (SP-D) have been found correlated with reduced survival (Greene et al., 2002; Maher et al., 2017), and serum levels of Krebs von den Lungen 6 (KL-6) have been shown to be associated with acute exacerbations of IPF and mortality (Ishikawa et al., 2012; Maher et al., 2017). Cancer antigens CA 19-9 and CA-125 have been shown to be elevated in patients with progressive disease (Maher et al., 2017). Genetic variants of MUC5B and TOLLIP have been associated with disease progression (Oldham et al., 2015). Thus, opportunities are emerging to allow for enriching clinical trials for patients at higher risk of IPF progression; however, none of the molecular biomarkers have yet been validated in larger clinical trials. Also, it is likely that not a single biomarker, but a biomarker signature will most reliably predict disease progression. Fewer data are available on possible therapeutic biomarkers; recent studies have demonstrated a short-term dynamic change in various putative biomarker analytes in response to therapeutics that might be predictive of a subsequent response to therapy (Nanthakumar et al., 2019; Jenkins et al., 2020; Luo et al., 2020). Further studies of such biomarker effects will be necessary but suggest the feasibility of biomarker-driven studies to improve clinical trial efficiency. In conclusion, biomarkers are not ready for prime time yet. They are increasingly being used to indicate target engagement or proof of clinical principle, but no biomarkers are available to substitute FVC as an endpoint in pivotal clinical studies in fibrosing interstitial lung diseases.

### Regulatory Requirements

The regulatory hurdles for registration of new drug candidates for IPF or fibrotic ILDs have been high in the past, requiring at least two replicate pivotal trials meeting their primary efficacy endpoint (i.e., decline in FVC) and providing acceptable safety over 1 year treatment duration. However, some light at the end of the tunnel can be seen that authorities may be increasingly willing to deviate from these classical rigid requirements, given the remaining high unmet need in pulmonary fibrosis and the challenges with existing clinical endpoints. An example is the recent approval byUS FDA of tocilizumab, an IL-6 inhibitor, to slow the rate of FVC decline in systemic sclerosis-associated ILD. This FDA approval is based on data from a single phase III randomized controlled clinical trial in 210 SSc-ILD patients with supportive evidence coming from a similar preceding (phase II) trial. The phase III trial did not meet its primary endpoint change from baseline to week 48 in the modified Rodnan Skin Score, a standard outcome measure for skin fibrosis in systemic sclerosis (Khanna et al., 2020), but showed stabilization of lung function in participants who received tocilizumab (mean change −14 vs. −255 ml; mean difference 241 ml), similar to the effect observed in the phase II trial which also missed its primary endpoint (Khanna et al., 2016). This recent approval indicates acknowledgement by the FDA of shortcomings and difficulties of available clinical endpoints for SSc-ILD, and that robust evidence may even by proven by consistent and substantial treatment differences in secondary outcomes such as FVC used in these studies.

## Conclusion

Pulmonary fibrosis is believed to result from aberrant repair of repeated epithelial injury. Application of new technologies has shifted our research focus beyond fibroblast biology into the immune-fibrotic niche and its complex dysregulated interplay of diverse cell populations. Differential patient biology using cell systems and tissue directly from patients and patient-relevant stimuli, as well as enhanced *in vivo* animal models reflecting the fibrotic niche, has become the starting point for precision drug discovery. Clinical development of new therapeutics for the treatment of rare fibrotic lung conditions is hampered by e.g., patient heterogeneity and the lack of appropriate biomarkers to predict the disease trajectory for the individual patient to enable enrichment strategies. This imposes major challenges on clinical trial design and project development timelines. These will hopefully be overcome by improved and efficient trial designs which are accepted by regulatory authorities and ultimately pave the way to precision medicine approaches to provide the right treatment at the right time to the right patient suffering from pulmonary fibrosis.
